# Antibiotic resistance patterns of *Staphylococcus* spp. isolated from fast foods sold in different restaurants of Mymensingh, Bangladesh

**DOI:** 10.5455/javar.2021.h512

**Published:** 2021-06-23

**Authors:** Monami Rahman Urmi, Wahedul Karim Ansari, Md. Saiful Islam, Md. Abdus Sobur, Marzia Rahman, Md. Tanvir Rahman

**Affiliations:** 1 Department of Microbiology and Hygiene, Faculty of Veterinary Science, Bangladesh Agricultural University, Mymensingh-2202, Bangladesh; 2 Department of Microbiology and Public Health, Faculty of Animal Science and Veterinary Medicine, Patuakhali Science and Technology University, Barisal, Bangladesh

**Keywords:** Antibiotic resistance, coagulase-positive, fast foods, MAR, MDR, public health, Staphylococcus aureus

## Abstract

**Objective:**

Fast foods are often responsible for staphylococcal foodborne illness. The present study was carried out to isolate *Staphylococcus* spp. from various fast foods sold in Mymensingh and to determine their antibiogram.

**Materials and Methods:**

Overall, 60 samples of fast foods sold in different restaurants were screened by culture, biochemical tests, and polymerase chain reaction (PCR) to isolate and identify *Staphylococcus* spp., followed by employing of disk diffusion method to determine their antibiotic resistance patterns.

**Results:**

Among these 60 samples, 8 [13.33%, 95% confidence interval (CI): 6.91%–24.17%] were positive for *Staphylococcus* spp. by cultural and biochemical properties. By PCR, four (6.67%, 95% CI: 2.62%–15.93%) isolates were confirmed as *Staphylococcus aureus *by targeting *nuc* gene. Additionally, all the *S. aureus* isolates were coagulase-positive. By antibiogram profiles, all the *Staphylococcus* isolates exhibited resistance to azithromycin and erythromycin (95% CI: 67.56%–100.00%), and frequently resistance to cefixime (75%, 95% CI: 40.93%–95.56%), ampicillin (50%, 95% CI: 21.52%–78.48%), and amoxicillin (37.5%, 95% CI: 13.68%–69.43%); moderate to lower resistance was found against cefotaxime, gentamicin, and doxycycline. In addition, all the isolates were sensitive to ciprofloxacin and chloramphenicol. Interestingly, 75% (6/8; 95% CI: 40.93%–95.56%) isolates were multidrug-resistant (MDR) in nature. Furthermore, the indices of multiple antibiotic resistance (MAR) were ranged from 0.2 to 0.6.

**Conclusion:**

This study revealed that fast foods sold in restaurants were contaminated with MDR and MAR *Staphylococcus* isolates, having potential public health significance.

## Introduction

Foods are edible or portable substances consumed by humans to sustain body growth and functions through energy generation. Fast foods are termed as meals that need minimal time to prepare, are served quickly to consumers in restaurants, and can be stored as precooked or preheated ingredients for further serving [1]. The consumption of fast foods is being increased globally due to its low price, ready form, and fit in any lifestyle. In Bangladesh, the number of fast food premises increases with the changes in societal life, employment systems, working times, and educational systems [2]. However, excessive consumption of fast foods is linked with different serious illnesses in humans, including obesity, heart attacks, strokes, psychiatric disorders, cancer, diabetes, dementia, etc. [3].

In Bangladesh, various fast foods are available, including burgers, vegetable rolls, chicken rolls, pastry cake, pizza, chicken toast, sandwiches, bread, egg, petis, etc. [4]. Gradually, fast food items are getting popularity among all classes of people. With this perspective, a significant number of ready-to-eat fast food shops are getting set up without concerning any kind of microbiological hygiene and safety [5]. Usually, fast food producers and handlers are poor and uneducated; thus, they have little or no knowledge on safe food management, hygienic food handling, display, and service [6]. Even they have no knowledge on safe raw materials and usage of potable water during food processing and manufacturing. As a result, fast foods can easily be exposed to microbial contamination, which can develop foodborne illnesses.

The foodborne illness developed by microorganisms is of major public health problem [7]. Most foodborne illnesses are linked with *Salmonella* spp., *Staphylococcus aureus*, *Campylobacter* spp., *Escherichia coli*,* Listeria monocytogenes*, *Clostridium perfringens*, *Toxoplasma gondii*, and others [8]. Bangladesh is one of the least developed countries, and the food inspection and legislative intervention of marketing low-quality food are very poor. As a result, poor-quality foods are readily available, indicating the higher risk of foodborne infections in Bangladesh.

Foodborne illnesses developed by *Staphylococcus* spp. are most common around the globe [9]. In addition, the consumption of *S. aureus*-contaminated foods is the leading cause of staphylococcal food poisoning (SFP) [7]. Furthermore, it becomes detrimental because of its zoonotic nature [10]. SFP is generally linked with poultry and poultry products, meat and meat products, bakery products (pizza, burger, pastry, bun, etc.), and salads [11]. The presence of this pathogen causes a great hazard for consumers and imposes economic losses via foodborne diseases. Nausea, abdominal cramps, vomiting, and even diarrhea are common manifestations of SFP [12]. Most of the staphylococcal infections are caused by *S. aureus*, a leading pathogen responsible for food poisoning and skin infections, pneumonia, endocarditis, and toxic shock syndrome in humans [13].

Antibiotic resistance is the most controversial topic in today’s world. The global population may face massive mortalities and severe economic losses due to the overuse of antibiotics [14]. Excessive use of antibiotics triggers selective pressure to develop antibiotic resistance among animals and humans [15,16]. The resistant bacteria and other pathways can transmit to humans through contaminated food consumption originating from animals [17]. The most severe types of drug-resistant *Staphylococcus* spp. are methicillin-resistant *S. aureus* and vancomycin-resistant *S. aureus* [18]. The development of multidrug-resistant (MDR) and multiple antibiotic resistance (MAR) creates severe problems in treating staphylococcal infections.

Organizations such as Bangladesh Standards and Testing Institution, Bangladesh Council of Scientific and Industrial Research, etc., have been established to standardize, test, certify, and grade the quality and marketing of different commodities and foods [19]. Nevertheless, a lot of food-producing industries have developed without taking concern from the respective authorities. For this reason, peoples are not getting wholesome food or food products always. In this situation, it is pivotal to monitor the microbiological checkup of fast food items and the related environment of each batch or lot circulating into the market. Therefore, the present study was carried out to isolate *Staphylococcus *species from fast foods sold in different restaurants within Mymensingh and determine their MDR and MAR profiles.

## Materials and Methods

### Ethical statement

No ethical approval was needed and verbal permission was taken from restaurant owners during sample collection.

### Sampling

**Figure 1.**
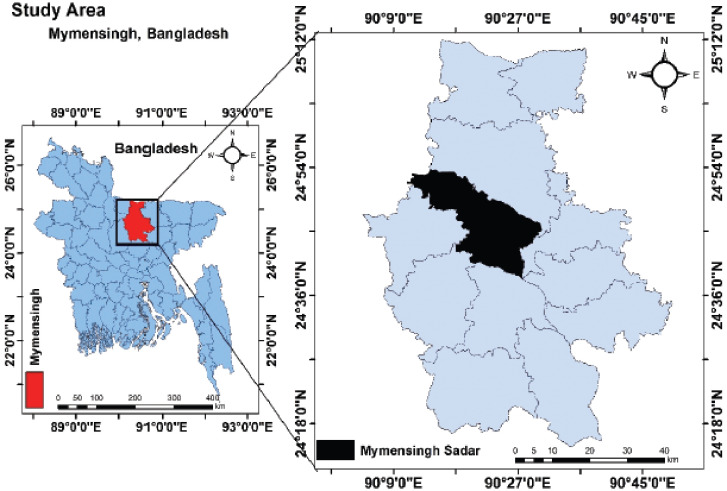
Study area map of the present study. The map was created with ArcMap (version 10.7) software (ESRI, Redlands, CA).

This study was carried out in the Ganginarpar, KR market, and Sheshmor market within the Mymensingh Sadar Upazila (24.7851°N, 90.3560°E) of the Mymensingh district of Bangladesh. The sampling site is shown in Figure 1. A total of 60 fast food samples of different preparations were aseptically collected. Samples were taken into sterile zip lock bags with the tag number and transported to the Food Hygiene Laboratory of the Department of Microbiology and Hygiene, Bangladesh Agricultural University, Mymensingh, to carry out the bacteriological analysis.

### Isolation of Staphylococcus spp.

Preliminary isolation of *Staphylococcus* spp. was based on culture, Gram stain, and biochemical tests as described later. Media used for the culture were blood agar and Mannitol salt agar (MSA) (Oxoid, UK) media. Approximately 1 gm of sample was first inoculated into 5 ml of nutrient broth containing sterile test tube and incubated overnight at 37°C for selective enrichment. One loopful overnight growth culture was streaked on a 5% bovine blood agar (BBA) plate, followed by incubating at 37°C for 24 h. After subsequent culture on BBA plates, colonies with characteristic features were subcultured on MSA plates and incubated following previous conditions to obtain pure colonies. Presumptive *Staphylococci* colonies were then screened by Gram stain and biochemical tests [20].

### Molecular detection of S. aureus targeting nuc gene

The isolated *Staphylococci* were subjected to polymerase chain reaction (PCR) to detect *S. aureus* targeting *nuc* gene (F: 5'-GCG ATT GAT GGT GAT ACG GTT-3' and R: 5'-AGC CAA GCC TTG ACG AAC TAA AGC-3,) with 279 amplicon size [21]. For PCR, bacterial genomic deoxyribonucleic acid (DNA) was extracted by boiling method [22]. In brief, initially, 1 ml of overnight growth bacterial fresh culture was centrifuged at 5,000 rpm for 5 min, followed by discarding of the supernatant. Subsequently, a similar procedure was followed after mixing and vortexing with 1 ml of phosphate buffer solution (PBS). After that, 200 μl of PBS was added and vortexed, and the suspension was boiled and cooled for 10 min in each step. Finally, the suspension was centrifuged for 10,000 rpm for 10 min, and the supernatant was collected as genomic DNA for further analysis.

All the PCR reactions were carried out in a final volume of 25 μl containing12.5 μl of the master mixture (Promega, USA), 5 μl of genomic DNA template, 1 μl of each forward and reverse primer, and 5.5 μl of nuclease-free water. The thermocycling conditions of the PCR amplification as follows: initial denaturation at 95°C for 5 min; 30 cycles of denaturation at 95°C for 1 min, annealing temperature at 55°C for 45 sec and extension at 72°C for 1.5 min; and the final extension at 72°C for 10 min. The amplified PCR products were then subjected to electrophoresis in 2% agarose gel, stained with ethidium bromide, and finally audited under ultraviolet trans-illuminator.

### Antibiotic susceptibility test (AST)

The AST of all *Staphylococci* isolates was carried out following the disk diffusion method [23]. Ten antibiotics under seven classes were employed in this study, i.e., macrolides (azithromycin, 15 μg/disk; erythromycin, 15 μg/disk), penicillin (amoxicillin, 10 μg/disk; ampicillin, 10 μg/disk), fluoroquinolones (ciprofloxacin, 5 μg/disk), tetracyclines (doxycycline, 30 μg/disk), aminoglycosides (gentamicin, 10 μg/disk), amphenicols (chloramphenicol, 30 μg/disk), and cephalosporins (cefotaxime, 30 μg/disk; cefixime, 5 μg/disk). For AST, overnight growth bacterial cultures were streaked on MSA plates, and two to three characteristic colonies were suspended to sterile PBS to adjust with 0.5 McFarland standard unit. Then, the suspension was disseminated homogenously on Mueller-Hinton agar (Oxoid, UK) plates using sterile cotton buds. Finally, the pre-selected antibiotics were dispensed on them and incubated for 24 h at 37°C to obtain the result. The results (resistant, intermediate, and sensitive) were interpreted by following the guidelines of the Clinical and Laboratory Standard Institute [24]. An isolate showing resistance against three or more classes of antibiotics was MDR in nature [25]. In addition, the MAR index was enumerated by the following formula: MAR = *p*/*q*, here “*p*” is the number of antibiotics resistant to an individual isolate, and “*q*” is the total number of tested antibiotics [26].

### Statistical analysis

Data from this study were initially input into Microsoft Excel 2013 (Los Angeles, CA) and subsequently exported into the GraphPad Prism 8.4.2 (GraphPad Software, Inc.) for further analysis. The binomial 95% confidence interval (CI) was enumerated, followed by the Wilson/Brown Hybrid method [27].

## Results

### Occurrence of Staphylococcus spp. and S. aureus

**Figure 2.**
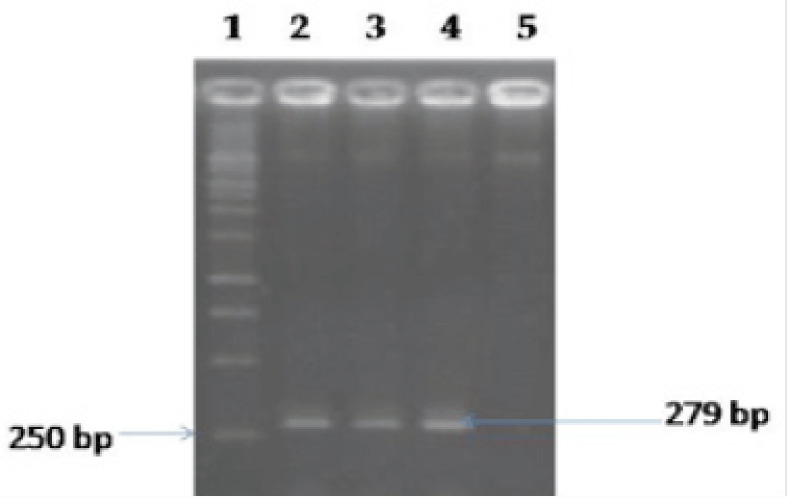
Representative PCR amplification of *S. aureus *targeting *nuc* gene. Lane 1, 1 Kb DNA ladder; Lane 2, positive control; Lane 3 and 4, representative *S. aureus* isolates; and Lane 5, negative control.

By cultural and biochemical tests, *Staphylococcus* spp. were found in 13.33% (8/60; 95% CI: 6.91%–24.17%) of fast food samples (Tables 1 and 2). Based on the presence of the *nuc* gene (Fig. 2), four fast food samples were found to be positive for *S. aureus*, denoting an occurrence of 6.67% (4/60; 95% CI: 2.62%–15.93%) (Tables 1 and 2). All four isolates were coagulase-positive of the *S. aureus* isolates (4/60; 95% CI: 2.62%–15.93%).

In area-wise, the highest occurrence of *Staphylococcus* spp. and *S. aureus* was exhibited in the Sheshmor market (13.33%) and KR market (6.67%) (Table 1).

For sample-wise, the highest occurrence of *Staphylococcus* spp. (20%) was detected in butter naan, sandwich, and pastry cake, and the highest detection rate of *S. aureus* was exhibited in pizza, sandwich, chicken roll, and pastry cake (Table 2).

### Antibiotic susceptibility test (AST) of Staphylococcus spp.

**Figure 3.**
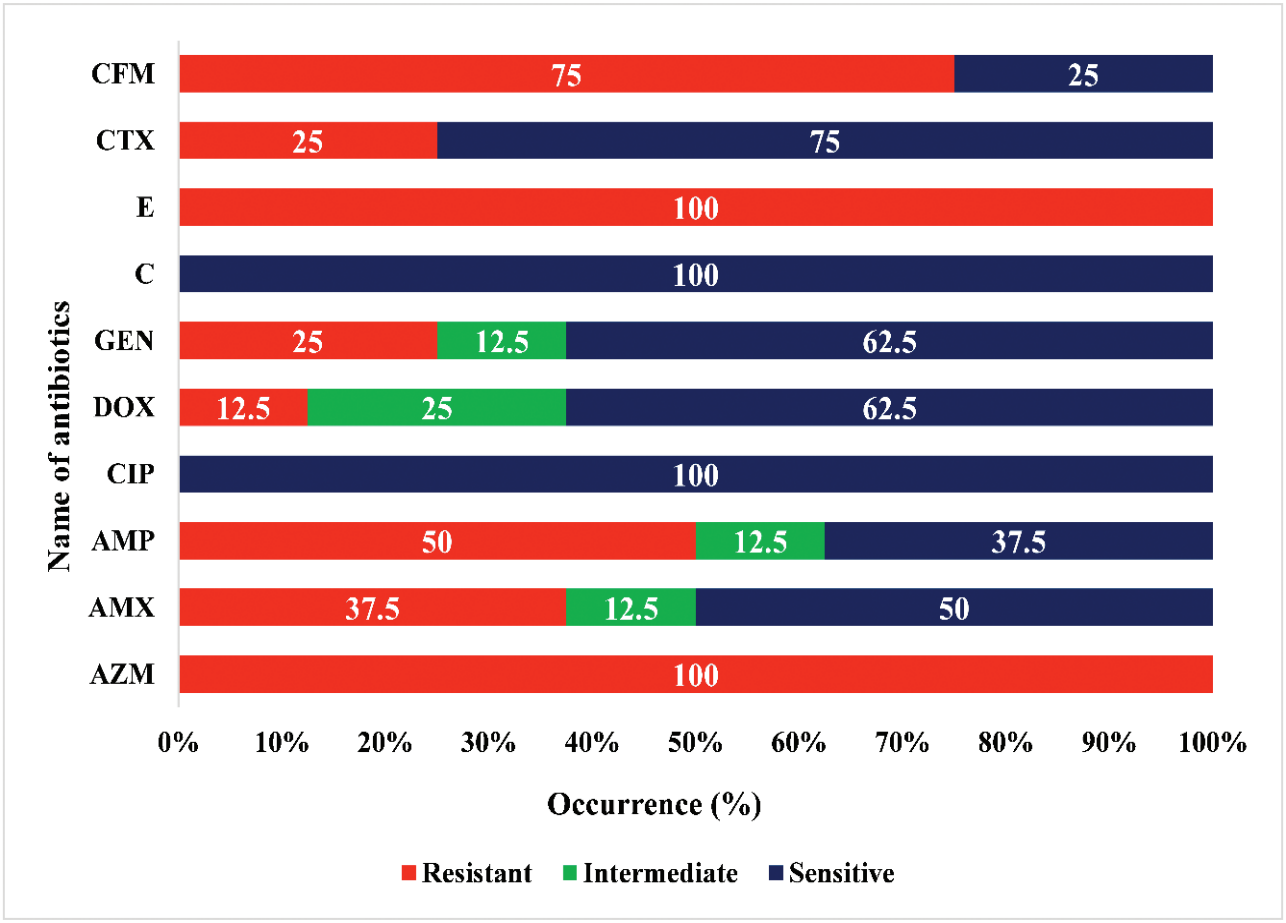
Antibiogram profiles of *Staphylococcus* spp. isolated from fast foods. AZM = azithromycin, AMX = amoxicillin, AMP = ampicillin, CIP = ciprofloxacin, DOX = doxycycline, GEN = gentamicin, C = chloramphenicol, E = erythromycin, CTX = cefotaxime, and CFM = cefixime.

The AST revealed that all the *Staphylococcus* isolates showed resistance to azithromycin and erythromycin (95% CI: 67.56%–100.00%), and frequently resistance to cefixime (75%, 95% CI: 40.93%–95.56%), ampicillin (50%, 95% CI: 21.52%–78.48%), and amoxicillin (37.5%, 95% CI: 13.68%–69.43%), followed by 25% (95% CI: 4.44%–59.07%) of cefotaxime and gentamicin, and 12.5% (95% CI: 0.64%–47.09%) of doxycycline. In addition, all the isolates were sensitive to chloramphenicol and ciprofloxacin. The overall antibiogram patterns are shown in Figure 3.

### MDR and MAR resistance profiles of Staphylococcus spp.

Among eight *Staphylococcus* isolates, six (75%, 95% CI: 95% CI: 40.93%–95.56%) were MDR in nature. Interestingly, all the isolates showed different antibiotic resistance patterns. Pattern 1 showed the highest number of antibiotics (six under four classes of antibiotics) were resistant to one *Staphylococcus* isolate (Table 2). In addition, all the *S. aureus* isolates were MDR in nature. Furthermore, the ranges of MAR observed in resistant *Staphylococcus* isolates were from 0.2 to 0.6. Among them, three isolates (37.5%, 95% CI: 13.68%–69.43%) contained 0.5 MAR index (Table 3).

**Table 1.** Occurrence of* Staphylococcus *spp. and *S. aureus *isolated from fast foods sold in different areas.

Name of area	No. of positive *Staphylococcus *spp. (%, 95% CI)	No. of positive *S. aureus* (%, 95% CI)
KR market (*n* = 45)	6 (13.33%, 6.26%–26.18%)	3 (6.67%, 2.29%–17.86%)
Ganginarpar (*n* = 10)	1 (10%, 0.51%–40.42%)	1 (10%, 0.51%–40.42%)
Seshmor market (*n* = 5)	1 (20%, 1.03%–62.45%)	0 (0%, 0.00%–43.45%)
Total (*n* = 60)	8 (13.33%, 6.91%–24.17%)	4 (6.67%, 2.62%–15.93%)

**Table 2.** Occurrence of *Staphylococcus* spp. and *S. aureus *isolated from different fast food samples.

Name of foods	Total	No. of positive *Staphylococcus *spp. (%, 95% CI)	No. of positive *S. aureus* (%, 95% CI)
Burger	10	1 (10%, 0.51%–40.42%)	0 (0%, 0.00%–27.75%)
Butter bun	10	2 (20%, 3.55%–50.99%)	0 (0%, 0.00%–27.75%)
Pizza	10	1 (10%, 0.51%–40.42%)	1 (10%, 0.51%–40.42%)
Sandwich	10	2 (20%, 3.55%–50.99%)	1 (10%, 0.51%–40.42%)
Chicken roll	10	0 (0%, 0.00%–27.75%)	1 (10%, 0.51%–40.42%)
Pastry cake	10	2 (20%, 3.55%–50.99%)	1 (10%, 0.51%–40.42%)

**Table 3.** Multidrug resistance and multiple antibiotic resistance profiles of *Staphylococcus* spp. isolated from fast foods.

Pattern no.	Antibiotic resistance pattern	No. of antibiotics (Classes)	No. of isolates	Overall MDR isolates	MAR index
1	AZM, AMX, AMP, GEN, E, CFM	6 (4)	1	6/8 (75%)	0.6
2	AZM, AMX, AMP, E, CFM	5 (3)	1		0.5
3	AZM, AMP, E, CTX, CFM	5 (3)	1		0.5
4	AZM, GEN, E, CTX, CFM	5 (3)	1		0.5
5	AZM, AMP, E, CFM	4 (3)	1		0.4
6	AZM, DOX, E, CFM	4 (3)	1		0.4
7^a^	AZM, AMP, E	3 (2)	1	-	0.3
8^a^	AZM, E	2 (1)	1	-	0.2

AZM = azithromycin, AMX = amoxicillin, AMP = ampicillin, DOX = doxycycline, GEN = gentamicin, E = erythromycin, CTX = cefotaxime, CFM = cefixime, MDR = multidrug-resistant, and MAR = multiple antibiotic resistant.

a Non-multidrug-resistant.

## Discussion

Fast foods are regarded as highly demandable foods globally due to their good flavor, reasonable price, and convenient handling. However, these food items are at risk of microbial contamination with different antibiotic-resistant and pathogenic microorganisms having serious public health significance. Therefore, the present study was designed to detect *Staphylococcus* spp. from fast foods sold in different restaurants and determine their MDR and MAR patterns.

In the present study, 13.33% of the fast food samples were contaminated with *Staphylococcus* spp., based on their characteristic features on cultural media and in different biochemical tests. Previously, Haider et al. [28] isolated *Staphylococcus* spp. from a burger in Bangladesh. In our present study, the occurrence of contamination in fast food samples gives an image of insufficient food hygiene practices in food processing and manufacturing. Persons involved in food processing and manufacturing may play a significant role in the contamination of these fast foods. In addition, the fast foods might be contaminated with *Staphylococci* from their source.

Furthermore, soft fast foods might be linked with bacterial contamination because of their moisture characteristics and short shelf-life due to bacterial spoilage. Soft foods are usually bacteriologically unstable due to the bacteriological metabolic activities [29]. In the context of food processing and manufacturing in Bangladesh, unsanitary conditions are taught to be the main sources of these contaminations. *Staphylococcus* spp. in fast foods poses a health risk for human consumption, and this may exist in every stage of food chain supply from preparation to consumptions [30].

In this study, 6.67% of the samples harbored *S. aureus*, which is lined with the previous study conducted in Northern Jordan with an 8.3% occurrence rate [31]. In Bangladesh, Islam et al. [32] revealed that processed raw or ready-to-eat fast foods harbored 22% *S. aureus,* which is higher than our present study. In addition, several previous studies detected *S. aureus* with a diversified occurrence rate from different food-related samples [33-36]. The variations of these studies with our study might be due to the variations in geographical locations, sample size and types, hygienic management, and others. However, the presence of *S. aureus* in fast foods reveals a high risk to human health.

All the *S. aureus* isolates were coagulase-positive. The presence of coagulase-positive *Staphylococcus* (CPS) in fast food samples shows serious pathogenic potentials. Their presence in food samples might be largely due to the unwholesome practices of the food handlers. The CPS is linked with large-spectrum infections ranging from skin infection to life-threatening consequences [37]. They can develop cytotoxins that can damage cellular membranes and develop pathogenic mechanisms in humans and animals [37].

Antibiotic resistance is a global health issue. In this study, all *Staphylococci* are resistant to azithromycin and erythromycin and frequently resistant to cefixime, ampicillin, and amoxicillin. Previously, Gundogan et al. [38] recorded almost similar antibiotic resistance patterns. In *Staphylococci*, erythromycin resistance may occur due to the ribosomal modifications triggered by 23S rRNA methylase enzyme (mediated by *ermA*, *ermB*, and *ermC* genes) or due to active efflux of antibiotics triggered by ATP-dependent pump (mediated by *msrA* gene) [39]. Furthermore, penicillin and beta-lactam antibiotics have become resistant to *Staphylococci* by *blaZ* gene of the organism, which assists in hydrolyzing beta-lactam rings and inactive the beta-lactam antibiotics [40]. Notably, 75% of the *Staphylococcus* isolates were resistant to cefixime, posing a health risk to humans. Cefixime is an antibiotic under third-generation cephalosporin used in severe infections in humans [41]. However, further molecular studies should be taken on hand to follow-up the present outcomes in our study.

Infections developed by MDR and MAR microorganisms show severe human health significance; also they could develop life-threatening consequences [42]. MDR *Staphylococci* have been revealed as the most common health problem globally. Alarmingly, in our present study, 75% of the *Staphylococci* isolated from fast foods were MDR in nature. In addition, the indices of MAR in *Staphylococcus* isolates ranging from 0.2 to 0.6 indicate that the antibiotics were used haphazardly in the source level of fast foods. In addition, it suggests that the processing or manufacturing of these fast foods were carried out under severe unhygienic conditions and were exposed to antibiotic-resistant microorganisms. The presence of MDR and MAR *Staphylococci* in fast foods reveals a severe public health hazard.

## Conclusion

The findings from the present study reveal that fast foods sold in different restaurants of Mymensingh are contaminated with pathogenic MDR and MAR *Staphylococcus* spp., which pose a potential health risk to humans. Training programs on food safety and managing foodborne diseases should be accomplished to the fast food vendors to minimize infection risk and get safe food among all facilities. In addition, consumers should be aware of the foodborne diseases and unhygienic conditions of fast foods. The preeminent step should be the systemic and structured monitoring programs implemented by the country’s healthcare authorities with strict law and regulations to ensure the safety of any kind of food.

## List of Abbreviations

AST: antibiotic susceptibility test; BBA: bovine blood agar; CI: confidence interval; CPS: coagulase-positive *Staphylococcus*; h: hour; MAR: multiple antibiotic resistance; MDR: multidrug-resistant; min: minute; MSA: Mannitol salt agar; PBS: phosphate buffer solution; PCR: polymerase chain reaction; sec: second; SFP: staphylococcal food poisoning.
